# Uremia

**Published:** 1883-08-20

**Authors:** J. H. Low

**Affiliations:** New York


### UREMIA?

BY J. H. LOW, M. D., OF NEW YORK.

The difficulties arising in forming a correct diagnosis, and some concomitant accompanying circumstances attending a case that I was called to a few days ago, in company with Dr. Birkins, of this city, induces me to think, after our close investigation, may be of some interest to your numerous readers, more particularly in our arriving at a satisfactory diagnosis so difficult for us to determine.

Mrs. W., resident near Fifth avenue, in this city, aged 80 years and five months, was seen first by Dr. Birkins, on the 16th of July at 3:40 p. m., complained of intense pain in the head and back, had had a short while previous to the Doctor's arrival a convulsion, and a short while after his arrival was seized with another convulsion, which she seemed not to rally from, and which ended in stupor.

At this time I first saw the patient. Her breathing was stertorous and heavy; her eyes partly opened but immovable, and insensible, one pupil contracted; circulation at radial artery full and about 90. I endeavored to arouse her by vigorous means, but failed in all my attempts, she appearing to be almost insensible. I supposed at first that she was laboring under the effect of some narcotic poison taken by accident or from suicidal motives (as there seems to be now prevailing a suicidal mania) but I was informed by the family that that was impossible as she had no chance of procuring opium or any poison. Being unable to administer remedies only by injection, we threw brandy and water into the rectum, applied sinapisms, etc., and at 10:25 a. m., on the 17th, we gave her a hypodermic injection of Magruder's solution of morphia of x drops. None of our efforts seemed to have the slightest effect. She gradually passed from the stupor to a deep-seated coma from which she never rallied, and died on the 19th of July.

Profuse diaphoresis obtained throughout the entire period of coma:

| TEMPERATURE. On the 17th. | PULSE. | RESPIRATION. | TIME. |
|---|---|---|---|
| 99½ | 86 | 40 | 1:20 A. M. |
| 98½ | 84 | 40 | 2:15 A. M. |
| 98½ | 84 | 38 | 3:15 A. M. |
| 98½ | 84 | 38 | 4:15 A. M. |
| 98¾ | 86 | 38 | 5:15 A. M. |
| 98½ | 86 | 38 | 9:30 A. M. |

After this there was no radial pulse perceptible. The axillary temperature, as a rule, was 2° higher than the ovum, and in the right axilla the temperature was 95°, in the left 99½°.

I proposed the application of electricity, but Dr. Birkins was fearful it might result, owing to her age and condition, in the rupture of some blood-vessel, so that was abandoned.

We tested her urine thoroughly and found a large quantity of albumen, and there was also a deposit of tube casts and epithelium.

We arrived at the following conclusions: Cause of death—Uræmia acute, developed in chronic Bright's disease, producing convulsions, stupor, coma and death.

Contributing causes: Old age, broken thigh and ulcer of six years standing.

